# Injury prevention and performance – are they mutually exclusive? What does science tell us?

**DOI:** 10.1186/2052-1847-7-S1-O7

**Published:** 2015-08-11

**Authors:** Alison McGregor

**Affiliations:** 1 Human Performance Group, Surgery & Cancer, Faculty of Medicine, Imperial College London, London, UK

## 

The academic journey into elite rowing performance at Imperial College has been evolving over the past 10-15 years. Its starting point was however, in injury prevention rather than performance enhancement, although this is clearly linked to performance. The major injury that drove this initial work was low back pain (LBP) and current research papers still noting that LBP accounts for 32% of reported injuries in elite rowers(Wilson et al 2010).

Injuries, particularly to the spine, have both immediate and longer term implications; the injury can not only prevent an athlete from competing, it can impair preparation and training and in some cases led to retirement from the sport. Furthermore, since rowing is predominately a team or crew based sport the impact of an injury goes beyond the individual athlete and can alter the subsequent success of the crew. It is not surprising therefore, that efforts to prevent injuries are important, however for athlete themselves injury prevention is perceived as less important since their focus is firmly on performance.

Rowing has been described as a motor skill that requires high levels of consistency, coherence, accuracy, and continuity. It is a highly repetitive action and thus the concept that lower back injuries are mechanical in origin and related to technique holds credence. By understanding how and why injuries occur we could impact on performance by preventing them as well as the long term well-being of the athlete. We have developed a system to measure the mechanics of the spine during rowing and have used this system to explore a situation that may precipitate injury including fatigue, training rate, experience etc. From this work we have been able to identify predictors of performance but experience derived from longitudinal measures suggest that some of these may also be important with respect to early identification of injury. However, as well as identifying injury it is important how to understand how to manage and change injury which in some instance may be related to technique and motor control. To this effect we will also explore the impact of biofeedback on performance and its use as a common language to facilitate change in rowing technique.
